# A gene expression predictor of response to EGFR-targeted therapy stratifies progression-free survival to cetuximab in KRAS wild-type metastatic colorectal cancer

**DOI:** 10.1186/1471-2407-9-145

**Published:** 2009-05-13

**Authors:** Justin M Balko, Esther P Black

**Affiliations:** 1 Department of Pharmaceutical Sciences, College of Pharmacy, University of Kentucky, Lexington, USA

## Abstract

**Background:**

The anti-EGFR monoclonal antibody cetuximab is used in metastatic colorectal cancer (CRC), and predicting responsive patients garners great interest, due to the high cost of therapy. Mutations in the KRAS gene occur in ~40% of CRC and are a negative predictor of response to cetuximab. However, many KRAS-wildtype patients do not benefit from cetuximab. We previously published a gene expression predictor of sensitivity to erlotinib, an EGFR inhibitor. The purpose of this study was to determine if this predictor could identify KRAS-wildtype CRC patients who will benefit from cetuximab therapy.

**Methods:**

Microarray data from 80 metastatic CRC patients subsequently treated with cetuximab were extracted from the study by Khambata-Ford et al. The study included KRAS status, response, and PFS for each patient. The gene expression data were scaled and analyzed using our predictive model. An improved predictive model of response was identified by removing features in the 180-gene predictor that introduced noise.

**Results:**

Forty-three of eighty patients were identified as harboring wildtype-KRAS. When the model was applied to these patients, the predicted-sensitive group had significantly longer PFS than the predicted-resistant group (median 88 days vs. 56 days; mean 117 days vs. 63 days, respectively, p = 0.008). Kaplan-Meier curves were also significantly improved in the predicted-sensitive group (p = 0.0059, HR = 0.4109. The model was simplified to 26 of the original 180 genes and this further improved stratification of PFS (median 147 days vs. 56.5 days in the predicted sensitive and resistant groups, respectively, p < 0.0001). However, the simplified model will require further external validation, as features were selected based on their correlation to PFS in this dataset.

**Conclusion:**

Our model of sensitivity to EGFR inhibition stratified PFS following cetuximab in KRAS-wildtype CRC patients. This study represents the first true external validation of a molecular predictor of response to cetuximab in KRAS-WT metastatic CRC. Our model may hold clinical utility for identifying patients responsive to cetuximab and may therefore minimize toxicity and cost while maximizing benefit.

## Background

A wealth of clinical data has confirmed the role of using KRAS mutational status to stratify advanced-stage colorectal cancer (CRC) patients to receive anti-EGFR monoclonal antibody (mAB) therapy [1-7]. Activating KRAS mutations are strong independent negative predictors of response to such treatment and mutational testing has been included in colorectal cancer practice guidelines. Interestingly, KRAS mutations may also predict lack of response to EGFR tyrosine kinase inhibitors (TKI) in lung cancer, suggesting a common mechanism of resistance to anti-EGFR therapies in these two tumor types [8-10]. Importantly, a large percent of lung cancer and CRC patients harboring wildtype KRAS, do not realize benefit from EGFR-targeted agents [1,3,5,7]. Therefore, additional methods of patient stratification are required to improve the tailoring of EGFR-targeted therapy in these diseases.

We have previously published a gene expression predictor of response (GEPR) to erlotinib in lung cancer [11]. The 180-gene model was built on Affymetrix microarray data and genes were selected and weighted based on the expression data from a series of lung cancer cell lines with known sensitivities to erlotinib. The model was externally validated using additional lung cancer cell lines as well as in human tumors (reference 11 and unpublished data). Given the correlation between KRAS mutational status and response to both EGFR-mAB and EGFR-TKI in lung and colorectal tumors, we hypothesized that our previously published GEPR is capable of predicting response to cetuximab in metastatic CRC.

Khambata-Ford and colleagues conducted a study with over 100 CRC patients wherein metastatic sites were biopsied, mutational status of KRAS was determined, and gene expression data was generated [12]. Following the biopsy, patients were treated with cetuximab as monotherapy and response and progression-free survival were recorded. The purpose of that study was to identify predictive biomarkers for response to cetuximab.

The publication of these data presented an excellent opportunity to test our hypothesis that the 180-gene GEPR to erlotinib generated in lung adenocarcinoma cell lines was portable to KRAS-wildtype CRC in predicting response to cetuximab. Since the data published by Khambata-Ford and colleagues was not available until almost a year following the publication of our predictive model, the data could be utilized to perform a true external validation, essentially equivalent to an independent prospective study due to the sequence and timing of the involved publications.

The primary endpoint of our study was to test the ability of our predictive algorithm to segregate cetuximab responders from non-responders in the KRAS-wildtype population included in the Khambata-Ford study. We found that our GEPR of erlotinib response was strongly predictive of cetuximab response with no gene-weighting adjustment or additional gene selection. However, reducing the signature to 26 of 180 genes based on the correlation of those genes to survival in the Khambata-Ford dataset significantly improved the predictive accuracy and Kaplan Meier curve separation. Importantly, the refined signature retained the original weights from the NSCLC model-training data, reducing the likelihood of over-fitting.

The most significant finding of this study was that the GEPR was capable of predicting progression-free survival in another tumor type than that on which the model was built, and with another EGFR-targeted agent. Similarly, other groups have previously reported portability of gene expression signatures [13,14]. We believe that this model could be highly useful in predicting response to cetuximab in CRC in patients with KRAS-wildtype tumors. Furthermore, additional studies to validate the predictive capacity of the model in other appropriate tumor types are underway.

## Methods

### Gene expression predictor of response to EGFR-targeted agents

The GEPR to EGFR-targeted agents was built using lung cancer cell lines and sensitivity data to the EGFR-tyrosine kinase inhibitor erlotinib. Briefly, the GEPR uses the MAS5-normalized Affymetrix signal intensity values from 180 genes which are represented on the Affymetrix U133 platform. These features are used to perform diagonal linear discriminant analysis (DLDA) in order to make a group selection of 'sensitive' or 'resistant' based on the similarity of the test sample to the 'sensitive' and 'resistant' training (model) data. The details regarding gene selection, weighting, and methods required to perform the analysis are reported elsewhere [11]. All predictive analyses for this study were carried out in R statistical language.

### Data analysis and prediction

The data from Khambata-Ford et al were extracted in series matrix format from Gene Expression Omnibus (GEO) record GSE5851[12]. The data from that study were scaled by the authors to mean intensity of 1500. Therefore, the data matrix was multiplied by a factor of 0.333 in order to reflect the mean intensity value of the data used to generate our GEPR (500). This direct linear relationship was confirmed by scaling experimental data to both values using Expression Console (Affymetrix, Santa Clara, CA) and observing the ratios on a probe by probe basis. A ratio of precisely 0.333 was observed for all probesets, confirming the validity of this approach to data handling (data not shown). The clinical response data and KRAS status were extracted from the supplementary files provided by Khambata-Ford et al [12].

No changes were made to the predictive algorithm. The test matrix was truncated to the 180 predictive genes included in the original model and imported into R for DLDA. Details regarding the analysis and model have been previously reported [11]. Data from the Khambata-Ford study were separated into three datasets: KRAS-wildtype, KRAS-mutant, or all patients combined. After sensitivity prediction on each of the datasets, the results were imported into Excel (Microsoft, Redmond, WA) and cross referenced with response and progression free survival (PFS).

### Model refinement

The 180 signature genes were filtered for their correlation to survival in the Khambata-Ford dataset. Specifically, ratios of gene expression values were calculated for the best (> 150 days PFS) relative to the worst-performing patients (< 50 days PFS). These cutoffs were selected based on the finding that they produced similarly sized cohorts of patients (approximately 1/3 of the dataset). These cohorts were used only to determine directionality of the genes (i.e. up in better-responding or down in better-responding). Similar ratios were calculated for each gene for the sensitive relative to resistant training (NSCLC) dataset. Next, genes which did not display directional concordance were filtered (i.e. genes with calculated ratios in both datasets > 1 or calculated ratios in both datasets < 1 were retained). This filtering step was performed because genes which show an association with response in the test dataset, but in the opposite direction, could confound the predictive model and are therefore not likely to improve the accuracy of the test. The remaining genes were further filtered based on their absolute correlation to PFS in the Khamabata-Ford dataset. Twenty-six of the original 180 genes were identified that had absolute Pearson's correlation coefficients of ≥ 0.2 and had directional concordance with the NSCLC model-training data. These 26 features were retained in the 'refined' model. This procedure was utilized only as a gene-filtering step, as the original weighting for these genes in the predictive algorithm was retained.

### Statistical analysis

All statistical analyses were performed using Prism (Graphpad, La Jolla, CA) and checked using JMP (SAS, Cary, NC). For comparisons of median progression free survival, the 2-tailed Mann-Whitney U test was performed between groups predicted to be sensitive and those predicted to be resistant. Kaplan-Meier survival curves were generated based on the PFS data reported by Khambata-Ford et al and analyzed by the log-rank statistic [12]. These analyses were performed on only the KRAS-wildtype patient data first, and then repeated on all patient data as well as the KRAS-mutant population independently for comparison.

## Results

### The gene expression predictor of response to erlotinib also predicts response and disease control to cetuximab in mCRC

The 180-gene GEPR provides a model which weights genes based on the expression values determined from a panel of NSCLC cell lines stratified by their sensitivity to the EGFR inhibitor erlotinib. This model was applied to the metastatic CRC data from Khambata-Ford et al. The microarray data from 80 of the 110 patients enrolled in that study were available for analysis. Of these, 43 (53.8%) were confirmed wildtype and 27 (33.8%) had confirmed KRAS mutations. The KRAS status of the remaining 10 (12.5%) patients was not reported.

**Figure 1:**
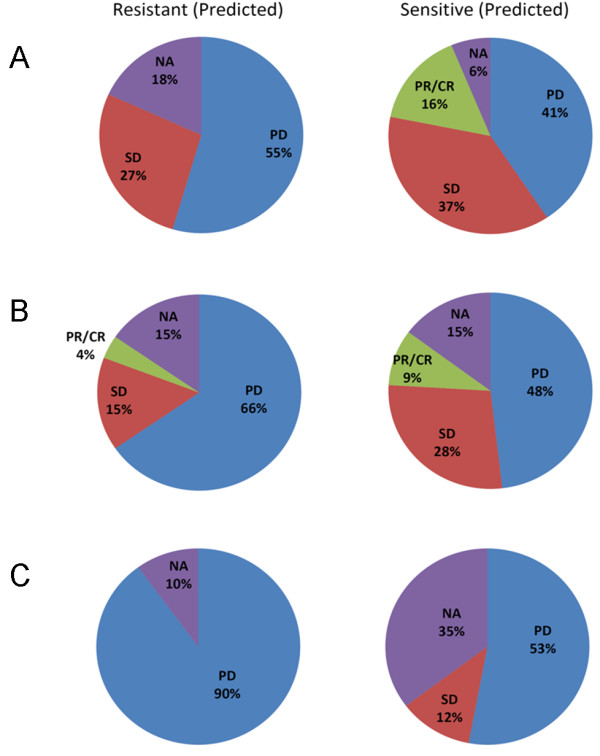
**Group classifications coupled with response data based on the 180-gene GEPR to EGFR-targeted therapy**. Patient populations from Khambata-Ford et al were classified using our predictive model. Percentage of patients exhibiting each annotated radiographic responses (CR/PR – complete responses and partial responses, SD – stable disease, PD – progressive disease, NA – not available/not reported) are classified as 'sensitive' or 'resistant' groups. A: KRAS-wildtype patients (43 subjects); B: all patients (80 subjects); and C: KRAS-mutant patients (27 subjects).

Since KRAS status is used to determine the CRC patients who should receive cetuximab, the primary endpoint of our study was to determine if the GEPR could correctly stratify KRAS-wildtype patients according to response/disease control. Indeed, when the prediction results were matched to response as reported by Khambata et al, our predictive model correctly captured 5/5 partial and complete KRAS-wildtype responders (Figure 1A). Also, 12/15 patients who demonstrated stable disease were classified as 'sensitive' by the GEPR. Thus, the majority (17/20, 85%) of patients demonstrating overall disease control (SD+PR+CR) were captured in the 'sensitive' group.

As a secondary endpoint, we wished to determine if the predictive signature was an independent predictor of response and/or disease control to cetuximab. To assess this, we analyzed all of the patient data, irrespective of KRAS status. Interestingly, when all 80 patients were considered, the GEPR retained predictive capacity (Figure 1B), although the most significant enrichment (Fisher's exact test, p = 0.001) of responding and SD patients were observed when the GEPR was applied only to KRAS-wildtype patients. No PRs or CRs were noted in KRAS-mutant patients, consistent with previous findings [2,5,7,12]. Nonetheless, 2/2 KRAS-mutant patients demonstrating stable disease to cetuximab were captured in the sensitive group (Figure 1C). Calculated parameters (specificity, sensitivity, negative predictive value, and positive predictive value) for the ability of the model to predict disease control are given in Table 1. Negative predictive values were consistently higher than positive predictive values, regardless of the cohort tested. This indicates that a high number of false positives are associated with this 180-gene model. However, in the absence of a genomic data to predict response, most wildtype KRAS mCRC patients could be considered candidates for cetuximab therapy. Therefore, the false positive rate is less concerning. The high NPV of the test suggests that the test could be accurately used to identify patients who will not benefit from anti-EGFR treatment (negative screen).

**Table 1:** Calculated parameters for the ability of the GEPR to predict disease control

Parameter	KRAS-wildtype	All patients	KRAS-mutant
Specificity	0.32	0.4	0.5
Sensitivity	0.85	0.8	1
PPV	0.57	0.43	0.18
NPV	0.66	0.77	1

NPV: negative predictive value; PPV positive predictive value

### The gene expression predictor of response to erlotinib stratifies cetuximab-treated mCRC patients based on progression-free survival

**Figure 2:**
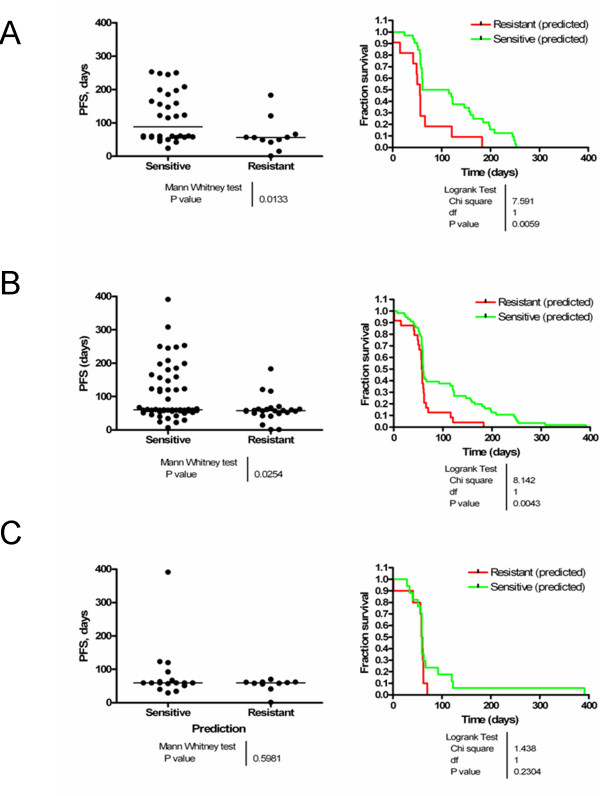
**The 180-gene GEPR to EGFR-targeted therapy stratifies a patient population demonstrating longer progression-free survival in metastatic CRC patients treated with cetuximab**. The data from Khambata-Ford et al were classified using our predictive model and matched to PFS. The scatter plots (left) depict the individual data points and median PFS for each group. The Kaplan-Meier survival curves (right) depict PFS between the 'sensitive' (green) and 'resistant' (red) groups. A: KRAS-wildtype patients; B: all patients; and C: KRAS-mutant patients.

We next determined whether KRAS-wildtype patients identified by the model as 'sensitive' would exhibit true clinical benefit when compared to those classified as 'resistant'. Scatter-plots and Kaplan-Meier survival curves of the progression-free survival (PFS) were generated for both predicted groups in KRAS-wildtype patients (Figure 2). PFS was significantly greater in the KRAS-wildtype patients who predicted as 'sensitive' (median PFS: 88 days, mean PFS: 117 days, 95% CI: 90.8 – 143.8 days) compared to those that predicted as 'resistant' (median PFS: 56 days, mean PFS: 63 days, 95% CI: 29.9 – 96.9 days) (Figure 2A, left). The difference in PFS was statistically significant between groups (p = 0.0133, two-tailed Mann-Whitney U test). The difference in the Kaplan-Meier survival curves was highly statistically significant when analyzed by the log-rank statistic for the KRAS-wildtype group (Figure 2A, right).

When the entire cohort was included in the analysis, regardless of KRAS status, the difference remained statistically significant (p = 0.0254, two-tailed Mann-Whitney U test) (Figure 2B, left). However, the differences in median and mean PFS were smaller (median PFS: 60 vs. 57.5 days and mean PFS: 104.7 vs. 60.5 days in 'sensitive' and 'resistant' subgroups, respectively). The difference in the Kaplan-Meier survival curves retained significance in the entire cohort, supporting the secondary hypothesis that the GEPR was an independent predictor of cetuximab benefit (Figure 2B, right). The PFS in the KRAS-mutant subgroup, when analyzed independently, was not statistically different between 'sensitive' and 'resistant' subgroups (Figure 2C).

### Refinement of the GEPR improves stratification of survival in KRAS-WT patients

**Figure 3:**
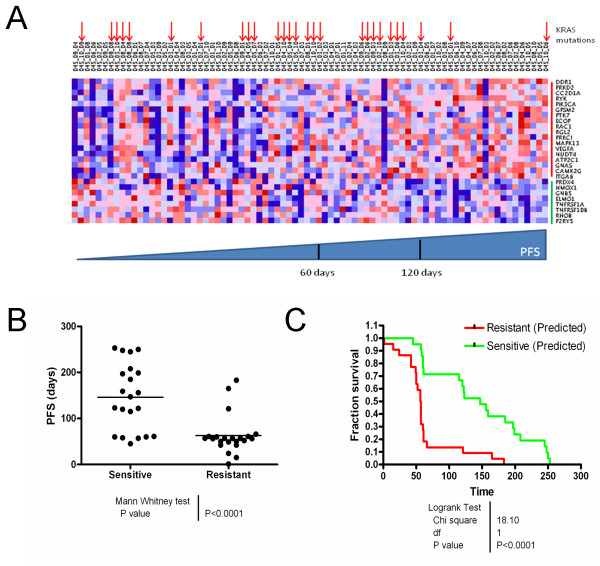
**Model refinement to 26 features improves predictive accuracy**. A. Heatmap of signal intensities for the 26 genes in the refined model plotted by clinical sample. All clinical samples were included (KRAS WT, KRAS-mutant, and unconfirmed/NA) and are arranged according to PFS. Sixty and 120 day PFS are identified on the scale for approximate reference. KRAS-mutant samples (codon 12) are designated by red arrows. Genes which are more highly expressed in the 'sensitive' training data are marked with red on the probeset axis, while gene more highly expressed in the 'resistant' training data are marked in green. B. The confirmed KRAS-WT samples were plotted by predicted sensitivity. Closed circles and bars represent individual PFS and median PFS for the group, respectively. C. The Kaplan-Meier survival curve depicts PFS between the 'sensitive' (green) and 'resistant' (red) groups. The reported p-value is for the log-rank statistic.

The 180-gene GEPR was effective in stratifying PFS in KRAS-WT patients. However, we wished to determine if the model features, which had been pre-defined, could be reduced to a suitable number for an alternate analytical method, such as qRT-PCR. Furthermore, data reduction could eliminate noisy genes, or those from the original model that are predictive in NSCLC but not predictive in colorectal cancer. To test this hypothesis, we filtered the 180 genes based on their directional concordance between the training data (NSCLC cell lines) and the CRC data. Genes that were more highly expressed in the sensitive NSCLC cell lines relative to the resistant lines while also being more highly expressed in CRC patients with a PFS of > 150 days were retained. Similarly, genes that were more highly expressed in the resistant NSCLC cell lines relative to the sensitive cell lines while also being more highly expressed in CRC patients with a PFS of < 50 days were retained. Finally, correlations of the remaining genes with PFS were calculated and those with an absolute value of correlation ≥ 0.2 were retained while those that were < 0.2 were filtered. A correlation cutoff of |0.2| returned a suitable number of genes and vastly improved differences in median survival between the two groups. A cutoff of |0.3| yielded only 8 genes, which we speculated may have a decreased likelihood of capturing tumor heterogeneity. Indeed, this signature did not effectively predict PFS in the dataset (data not shown). A cutoff of |0.1| yielded 75 genes, which we perceived to be a suboptimal data reduction step. This 75 gene signature improved predictions, but not as significantly as a cutoff of r = |0.2| (data not shown). Thus, twenty-six genes comprised the 'refined' model (Table 2). The weights for the 26 genes were retained from the original GEPR, to minimize over-fitting the model. A heatmap of the signal intensities of the 26 retained genes demonstrated a pattern of deregulation coincident with PFS (Figure 3A).

**Table 2:** Features of the 26-gene refined model

Gene	Description	Affymetrix Probe ID
DDR1	discoidin domain receptor tyrosine kinase 1	1007_s_at
PRDX4	peroxiredoxin 4	201923_at
RYK	RYK receptor-like tyrosine kinase	202853_s_at
HMOX1	heme oxygenase (decycling) 1	203665_at
GNB5	guanine nucleotide binding protein (G protein), beta 5	204000_at
PIK3CA	phosphoinositide-3-kinase, catalytic, alpha polypeptide	204369_at
ELMO1	engulfment and cell motility 1	204513_s_at
GPSM2	G-protein signaling modulator 2 (AGS3-like, C. elegans)	205240_at
PTK7	PTK7 protein tyrosine kinase 7	207011_s_at
TNFRSF1A	tumor necrosis factor receptor superfamily, member 1A	207643_s_at
ECOP	EGFR-coamplified and overexpressed protein	208091_s_at
RAC1	ras-related C3 botulinum toxin substrate 1 (rho family, small GTP binding protein Rac1)	208641_s_at
RGL2	ral guanine nucleotide dissociation stimulator-like 2	209110_s_at
TNFRSF10B	tumor necrosis factor receptor superfamily, member 10b	209295_at
PRKCI	protein kinase C, iota	209678_s_at
MAPK13	mitogen-activated protein kinase 13	210058_at
VEGFA	vascular endothelial growth factor A	210512_s_at
RHOB	ras homolog gene family, member B	212099_at
NUDT4	nudix (nucleoside diphosphate linked moiety X)-type motif 4	212181_s_at
ATP2C1	ATPase, Ca++ transporting, type 2C, member 1	212255_s_at
GNAS	GNAS complex locus	212273_x_at
CAMK2G	calcium/calmodulin-dependent protein kinase (CaM kinase) II gamma	212757_s_at
ITGA6	integrin, alpha 6	215177_s_at
P2RY5	purinergic receptor P2Y, G-protein coupled, 5	218589_at
PRKD2	protein kinase D2	38269_at
CC2D1A	coiled-coil and C2 domain containing 1A	58994_at

When the refined model was applied to the KRAS-WT CRC data, the resulting difference in PFS between the predicted-sensitive and predicted-resistant group was highly significant (median 147 days vs. 56.5 days in the predicted sensitive and resistant groups, respectively, p < 0.0001) (Figure 3B and 3C). Kaplan-Meier curves demonstrated significant separation as well. Parameters (sensitivity, specificity, NPV and PPV) for the refined model to predict disease control (SD/PR/CR) are presented in Table 3. Parameters were improved for the 26-gene model over the 180-gene model for all cohorts tested. Thus, as we have demonstrated, the utility of the model is greatly enhanced by enriching for genes which correlate with PFS.

**Table 3:** Calculated parameters for the ability of the refined (26-gene) GEPR to predict disease control

Parameter	KRAS-wildtype	All patients	KRAS-mutant
Specificity	0.74	0.71	0.78
Sensitivity	0.80	0.80	1.00
PPV	0.76	0.63	0.33
NPV	0.78	0.86	1.00

NPV: negative predictive value; PPV positive predictive value

## Discussion

The anti-EGFR monoclonal antibodies cetuximab and panitumumab are frequently used in metastatic CRC and improve overall survival when used in unselected populations [15-19]. However, a number of independent studies have elucidated the correlation of activating mutations in KRAS with lack of response to EGFR-targeted agents, and patient stratification based on KRAS status should improve overall survival through enrichment of responding patients [1-6]. However, a significant number of KRAS-wildtype patients do not benefit from treatment, and therefore additional methods to enrich the treated population for responders are needed to reduce unnecessary toxicity and cost while maximizing therapeutic benefit from these agents. Indeed, Karapetis and colleagues reached the conclusion that additional biomarker approaches are needed to identify KRAS-wildtype patients who will receive benefit from cetuximab in one of the largest analyses to date of the association of KRAS status with clinical outcome to cetuximab in CRC [7].

In this study, we utilized a GEPR for erlotinib, an EGFR-TKI, which was generated in lung cancer cell lines, to test its predictive capacity in KRAS-wildtype mCRC patients treated with the anti-EGFR mAB cetuximab. It is important to note that the GEPR generated in lung cancer cell lines and was not dependent on either KRAS or EGFR mutation status. Further, the genes included in the signature demonstrate biological association with pathways downstream of EGFR, including both the PI3K/AKT and MAPK pathways [11].

Application of our model to the CRC dataset represents a true external validation of the GEPR since the validation set was not available until well after the reporting of our GEPR model. The availability of this dataset allowed us to determine whether the GEPR could predict response to alternate EGFR-targeted agents, employ the use of KRAS status to enrich the predictive power, and function across tumor types (CRC versus non-small cell lung) [11,12]. Surprisingly, the unaltered 180-gene model had a high capacity to stratify KRAS-wildtype CRC patients who demonstrated disease control or response to cetuximab treatment. The data were furthered by the significant separation of the survival curves of the predicted 'sensitive' group versus the predicted 'resistant' group.

Importantly, these results were achieved even though the genes that comprised the model were selected and weighted based on the genomic expression in lung cancer cell lines. Unlike the data reported by Khambata-Ford and colleagues, neither amphiregulin (AREG) nor epiregulin (EREG) are included in our GEPR. Further, RNA isolation from biopsy of metastatic CRC of unknown tumor cell content and subsequent microarray hybridizations were all performed at a different facility than our own.

In the original report, Khambata-Ford and colleagues used AREG and EREG expression to stratify KRAS-wildtype patients, and found a significant improvement in PFS in the 'high' ligand expressers group (EREG: P = .0002, hazard ratio [HR] = 0.47, and median PFS, 103.5 v 57 days, respectively; AREG: P < .0001, HR = 0.44, and median PFS, 115.5 v 57 days, respectively)[12]. The differences in median survival reported in that study are greater than those identified in our study using the original 180-gene model. It is not surprising that the authors were able to demonstrate separation of the survival curves between high ligand expressers and low ligand expressers because AREG and EREG were chosen as biomarkers post-hoc. AREG and EREG were selected from over 600 genes after the response and progression free survival in the study population was already determined. Optimal cutoff expression levels were obtained from a receiver-operator characteristic (ROC) curve, and changes in median PFS were then calculated on the same data used to generate these variables. It has yet to be shown whether AREG and EREG hold any external validity as predictors of cetuximab response. In contrast, our predictive model was generated prior to the reporting of the Khambata-Ford data and using these data, provides true external validity of our model. The improvement in progression-free survival that we identified in the predicted 'sensitive' KRAS-wildtype mCRC patients was approximately 1 month. Given that cetuximab yields an overall benefit in PFS of 1.5 months as monotherapy in CRC as well as the high cost of treatment, these findings should be considered clinically important [20].

In light of the cost associated with microarray analysis, we went on to attempt to reduce the number of predictive genes necessary to achieve both response prediction and PFS stratification using data from the Khambata-Ford et al study. In so doing, we found that refining the GEPR, using a subset of 26 of the original 180 genes, greatly improved the sensitivity and specificity of the GEPR. Furthermore, using the refined 26-gene GEPR significantly improved the difference in median PFS between the predicted-sensitive and predicted-resistant groups and resulted in improved predictions compared with those reported by Khambata-Ford et al. Distinct differences in the gene expression patterns are observed in the gene expression values (color scheme of the heat map in Figure 3) of the 26 feature signature, clearly identifying a trend which corresponds to PFS. However, the variability of these patterns observed on a per gene basis highlights the necessity of using multiple features to capture the heterogeneity of tumors. As with the Khambata-Ford analysis, careful interpretation of the predictive accuracy of our refined model is necessary. Because information from the validation set was utilized in feature selection, over-fitting remains a possibility. The refined GEPR reported here retains the original weights of the 26 genes, reducing the chance of over-fitting. Additional validation will test that hypothesis and determine if the 26-gene GEPR can be used in a qRT-PCR analysis rather than on an Affymetrix platform.

While the 180-gene GEPR was useful for stratifying KRAS-wildtype patients, we also wished to determine whether the GEPR could stratify patients independently of KRAS status. Statistical significance was retained in both median PFS and the log-rank analyses when patients were not stratified based on KRAS status, suggesting that the signature is an independent predictor of benefit to cetuximab therapy in mCRC. However, patients with KRAS-mutant CRC tumors who predicted as 'sensitive' did not have longer PFS than those who predicted as 'resistant', although this could be due to the small sample size included in this particular analysis. It is of note that one patient with a KRAS-mutant tumor was reported by Khambata et al to have had a PFS of > 1 year on cetuximab, although radiographic response in this patient was not recorded. Our 180-gene GEPR classified this patient as 'sensitive', offering additional support of the independency of our test from KRAS mutational status. However, a significant number of non-responding KRAS-mutant patients were called 'sensitive' by the GEPR, contributing to a poor positive predictive value in this group.

To further explore the relationship between the GEPR prediction status and KRAS status, we performed a χ^2 ^test. No association with KRAS status was found in the prediction outcomes for either the 26-gene (p = 0.2) or the 180-gene signature (p = 0.3). Thus, our test appears to be independent of KRAS status. On a per gene basis, we also examined whether any of the 180 genes were significantly different between the KRAS-wildtype and KRAS-mutant cohorts. Of the 180 genes, 32 were p < 0.05 according to a two tailed t-test (although a Bonferroni correction yielded no significantly deregulated genes). However, only 3 of these genes were included in the final 26 gene signature (ATP2C1, P2RY5, and TNFRSF10B). Thus, this test offers an explanation for why the 26-gene signature demonstrated improved predictive accuracy over the 180-gene signature, as the majority of genes associated with KRAS activation appear to have been removed during gene list filtering.

Our GEPRs, 180- or 26-gene, may be best utilized in tandem with KRAS-mutational testing. Importantly, our methodology could easily be combined with KRAS mutational testing through biopsy of metastatic sites and allotment of tissue cores for both RNA and DNA purification. The high sensitivity and negative predictive value of the test suggests that use of the model could be implemented to significantly enrich the responding patient population while minimizing the number of potential-responders (i.e. false negatives) who would be diverted from receiving cetuximab.

## Conclusion

These data suggest that the 180-gene GEPR will be a valuable clinical tool in determining who should receive cetuximab therapy in metastatic colorectal cancer, perhaps best used in combination with KRAS status. More studies will be necessary to determine whether the predictive capacity of the model is retained in patients treated with cetuximab plus chemotherapy or in patients treated with panitumumab. Additional validation in NSCLC and CRC, and potentially other epithelial tumor types, will confirm the broader clinical utility of this predictive model, as well as assess the true external validity of the refined 26-gene model.

## List of abbreviations

mAB: monoclonal antibody; TKI: tyrosine kinase inhibitor; EGFR: epidermal growth factor receptor; PFS: progression free survival; GEPR: gene expression predictor of response; SD: stable disease; PD: progressive disease; PR: partial response; CR: complete response; NPV: negative predictive value; PPV: positive predictive value; NSCLC: non small cell lung cancer; CRC: colorectal cancer.

## Competing interests

JM Balko and EP Black are pursuing patent protection on the use of the predictive method reported here.

## Authors' contributions

JMB co-authored the manuscript and performed the data analysis. EPB provided scientific direction and co-authored the manuscript.

## Pre-publication history

The pre-publication history for this paper can be accessed here:

http://www.biomedcentral.com/1471-2407/9/145/prepub
